# Towards Immunotherapy-Induced Normalization of the Tumor Microenvironment

**DOI:** 10.3389/fcell.2022.908389

**Published:** 2022-05-30

**Authors:** Vinicio Melo, Edwin Bremer, John D. Martin

**Affiliations:** ^1^ Department of Hematology, University of Groningen, University Medical Center Groningen, Groningen, Netherlands; ^2^ Materia Therapeutics, Las Vegas, NV, United States

**Keywords:** tumor microenvironment, vascular normalization, immune checkpoints, hypoxia, immunotherapy, angiogenesis, immune cell infiltrate

## Abstract

Immunotherapies modulate the function of immune cells to eradicate cancer cells through various mechanisms. These therapies are successful across a spectrum of cancers, but they are curative only in a subset of patients. Indeed, a major obstacle to the success of immunotherapies is the immunosuppressive nature of the tumor microenvironment (TME), comprising the stromal component and immune infiltrate of tumors. Importantly, the TME in most solid cancers is characterized by sparsely perfused blood vessels resulting from so-called pathological angiogenesis. In brief, dysregulated development of new vessels results in leaky tumor blood vessels that inefficiently deliver oxygen and other nutrients. Moreover, the occurrence of dysregulated fibrosis around the lesion, known as pathological desmoplasia, further compresses tumor blood vessels and impairs blood flow. TME normalization is a clinically tested treatment strategy to reverse these tumor blood vessel abnormalities resulting in stimulated antitumor immunity and enhanced immunotherapy efficacy. TME normalization includes vascular normalization to reduce vessel leakiness and reprogramming of cancer-associated fibroblast to decompress vessels. How immunotherapies themselves normalize the TME is poorly understood. In this review, we summarize current concepts and progress in TME normalization. Then, we review observations of immunotherapy-induced TME normalization and discuss the considerations for combining vascular normalizing and immunotherapies. If TME could be more completely normalized, immunotherapies could be more effective in more patients.

## Introduction

Cancer cells coopt the surrounding tissue resulting in an organ-like structure with abnormal physiology. Specifically, they can promote unrestrained angiogenesis (i.e., the formation of new vessels) and desmoplasia (i.e., the formation of new and excessive connective tissue). The extent of each process depends on the type of tumor. For example, hepatocellular carcinoma (HCC) is highly angiogenic, whereas pancreatic ductal adenocarcinoma is highly desmoplastic. Dysregulated angiogenesis produces leaky blood vessels while desmoplasia compresses them (Jain, 2014). Thus, both processes reduce the capacity of blood vessels to deliver oxygen to tumors through independent mechanisms (Stylianopoulos and Jain, 2013; Jain, 2014; Martin et al., 2016). Sub-physiological oxygen tension is referred to as hypoxia (Semenza, 2014; Vaupel and Mayer, 2014). Besides promoting disease progression (Bhandari et al., 2019) and resistance to radiation (Martin and Jain, 2020) and some chemotherapies (Teicher et al., 1981), hypoxia causes immunosuppression in the tumor microenvironment (TME) by altering immune cell phenotype, infiltration, migration, and function (Noman et al., 2015). Simultaneously, newly formed immature blood vessels cannot traffic and distribute infiltrating immune cells efficiently (Slaney et al., 2014), whereas excessive fibrosis poses a physical barrier to immune cell migration into the tumor (Salmon et al., 2012). Accordingly, hypoxia is associated with poor survival across tumor types (Martin et al., 2019b) and alleviating hypoxia through ‘normalization’ of the TME increases the efficacy of immunotherapies in preclinical cancer models (Huang et al., 2012; Chauhan et al., 2019; Chen et al., 2019; Shigeta et al., 2019; Panagi et al., 2020; Shigeta et al., 2020; Mpekris et al., 2021; Voutouri et al., 2021; Mpekris et al., 2022). Circulating, tissue and imaging biomarker studies in patients with glioblastoma (Sorensen et al., 2009) and breast cancer (Tolaney et al., 2015; Boucher et al., 2021) support the notion that normalizing blood vessels with antiangiogenic therapies (AATs) correlates with increased antitumor immune cell infiltration and better treatment outcomes. Similarly, imaging studies in patients with lung cancer and glioblastoma indicate that increased blood flow (Sorensen et al., 2012; Heist et al., 2015) and reduced hypoxia (Batchelor et al., 2013) during AAT treatment correlated with response rates and overall survival. Furthermore, regimens of AATs combined with immune checkpoint inhibitors (ICIs) are approved by the United States Food and Drug Administration in patients with HCC, renal cell carcinoma, and non-small-cell lung cancer (Pinter et al., 2021). Whereas no benefit has been demonstrated in clinical trials directly comparing AAT and ICI versus ICI alone, the combination of bevacizumab with atezolizumab increased overall survival in patients with unresectable HCC compared to first-line treatment, sorafenib, which is a multikinase inhibitor with antiangiogenic properties (Finn et al., 2020). In contrast, ICI monotherapy did not increase overall survival compared to sorafenib (Finn et al., 2019; Yau et al., 2019). Thus, there is preclinical and clinical evidence supporting the notion that alleviating hypoxia through TME normalization increases immunotherapy efficacy. If we could understand how to better increase oxygen delivery when normalizing the TME for immunotherapy, then we might be able to improve outcomes for patients.

### The Impact of the Abnormal Tumor Microenvironment on Vessel Function

**FIGURE 1:**
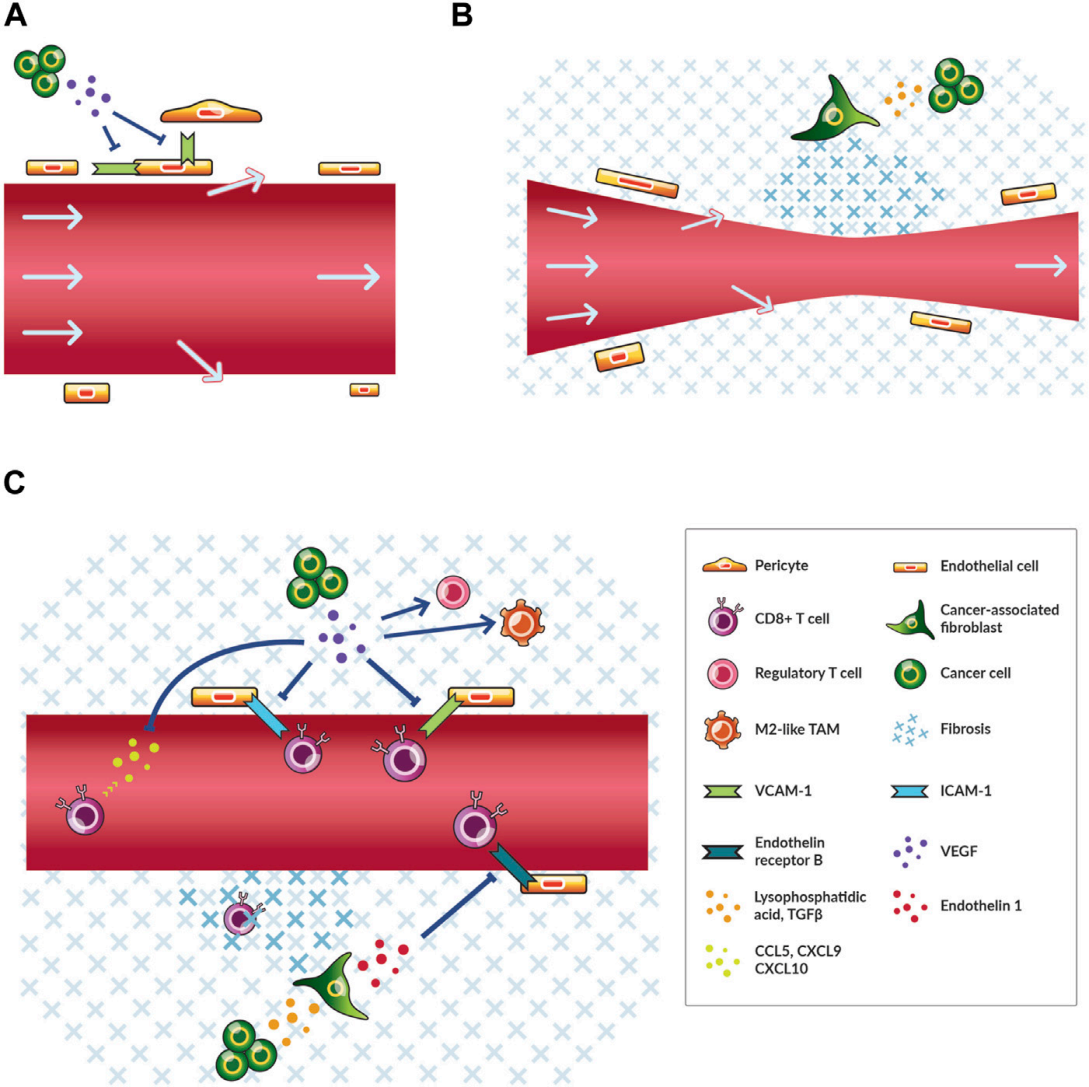
Cancer cells contribute to angiogenesis, desmoplasia, and immunosuppression thereby reducing blood flow and immune cell infiltration. **(A)** A simplified schematic of leaky tumor blood vessels and relevant cells. Cancer cells secrete angiogenic factors, including vascular endothelial growth factor (VEGF), that reduce the expression of molecules, such as intercellular adhesion molecule 1 (ICAM-1), which facilitate the interaction of endothelial cells with themselves and perivascular cells in normally functioning vessels. Plasma and blood-borne molecules (arrows) flow out of pores in the vessel wall thereby reducing flow. **(B)** A simplified schematic of compressed tumor blood vessels and relevant cells. Cancer cells activate cancer-associated fibroblasts through various signals including transforming growth factor (TGF) *β*. As a result, more fibrosis is produced and maintained thereby transferring compressive physical force onto blood vessels. Blood flow is reduced. **(C)** A simplified schematic of immunosuppression and relevant cells. As in **(A)** and **(B)**, cancer cells secrete VEGF and TGFβ among other factors that affect angiogenesis and fibrosis. VEGF recruits regulatory T cells and shifts tumor-associated macrophages (TAMs) towards M2-like immunosuppressive phenotypes. VEGF also blocks the recruitment and transmigration of CD8^+^ T cells. TGFβ signaling leads to increased fibrosis that physically impedes the migration of CD8^+^ T cells to cancer cells and blocks the vascular transmigration of these cells through endothelin one signaling through the endothelin receptor type **(B)**

Cancer cells induce nearby non-malignant cells to produce a microenvironment that promotes disease progression (Quail and Joyce, 2013; Bhandari et al., 2019; Thienpont et al., 2016) and immunosuppression (Chen and Mellman, 2017; Tauriello et al., 2018). As described above, the TME affects blood vessel formation through either disturbed angiogenesis or excessive fibrosis. The former process results from cancer cells sending signals to vascular, mesenchymal, and immune cells that impair physiological processes and blood vessel formation (De Palma et al., 2017; Fukumura et al., 1997). In brief, pathological angiogenesis is characterized by cancer cells stimulating endothelial and perivascular cells through angiogenesis and hypoxia signaling to produce new blood vessels through various mechanisms (Carmeliet and Jain, 2011a; Carmeliet et al., 1998; Fukumura et al., 1998). The main angiogenic signaling player in this context is vascular endothelial growth factor (VEGF), as it is released in response to hypoxia and triggers vascular cells to dissociate, migrate and remodel the surrounding tissue. These newly-forming tumor blood vessels do not mature because of constant pro-angiogenic VEGF signaling (Jain, 2003; Goel et al., 2011), which limits expression of integrins and cell adhesion molecules (Piali et al., 1995). The latter two are required for fortification of the mural cell (*i.e.*, pericytes and vascular smooth muscle cells) of tumor blood vessels (Garmy-Susini et al., 2005) (Figure 1A). Consequently, in the tumor the blood vessels are abnormal in shape and spatial distribution (Goel et al., 2011). Endothelial and mural cells become migratory, lose their interactions with each other (Hashizume et al., 2000; Morikawa et al., 2002; Baluk et al., 2003; Carmeliet and Jain, 2011b; Seano et al., 2014), and cannot adhere infiltrating immune cells (Slaney et al., 2014). As a result, blood vessels become leaky and are ineffective in maintaining blood flow, resulting in plasma (Boucher et al., 1990) and protein (Gerlowski and Jain, 1986; Stohrer et al., 2000) accumulation in the interstitial (i*.e.* extravascular) space (Figure 1A).

The second process of excessive fibrotic tissue formation is triggered by cancer cell-mediated activation of fibroblasts, which increases fibroblast contractility and production of fibrosis (Figure 1B). These cancer-associated fibroblasts (CAFs) have numerous phenotypes and also play a key role in immunosuppression (Sahai et al., 2020). Cancer cells and CAFs generate physical forces that compress tumor vessels (Stylianopoulos et al., 2012). Also, CAFs produce and maintain elevated levels of extracellular matrix (ECM) components (*i.e.,* structural components of the tissue including collagen I and hyaluronan) that transmit forces towards compressing blood vessels (Stylianopoulos et al., 2012; Chauhan et al., 2013; Chauhan et al., 2014) (Figure 1B). The expansion of a growing tumor is resisted by the surrounding host tissue thereby increasing the magnitude of compression exerted on tumor tissue (Stylianopoulos et al., 2013; Nia et al., 2016). Thus, leaky and compressed tumor blood vessels induce hypoxia.

Besides promoting hypoxia leading to immunosuppression, cancer cells suppress the activation, priming, trafficking, infiltration, migration, and function of antitumor immune cells to reduce antitumor immunity. In fact, all steps of the cancer-immunity cycle, which describes the processes that must be perpetuated for antitumor immunity, are subjected to negative regulation in the TME including through hypoxia signaling (Chen and Mellman, 2013; Martin et al., 2020). For instance, activated CD8^+^ T cells primed against cancer antigens must traffic to and infiltrate into tumors (Chen and Mellman, 2013). However, this process is inhibited by solid tumors (Slaney et al., 2014), with aberrant VEGF signaling downregulating cell adhesion molecules, such as intercellular adhesion molecule 1 (ICAM-1) and vascular cell adhesion molecule 1 (VCAM-1), thereby preventing activated CD8^+^ T cells to bind and cross the vessel wall (Piali et al., 1995; Griffioen et al., 1996) (Figure 1C). Additionally, a majority of immune cells in the TME are shifted to an immunosuppressive phenotype, such as M2-like rather than M1-like tumor-associated macrophages (TAMs) and regulatory rather than CD8^+^ T cells (De Palma et al., 2017) (Figure 1C). TAMs are shifted to M2-like phenotypes (Maenhout et al., 2014; Noman et al., 2015) and regulatory T cells are recruited through angiogenic and hypoxia-induced signaling (Facciabene et al., 2011; Voron et al., 2015; Wallin et al., 2016; Palazon et al., 2017). The TME can exist in different immune phenotypes that reflect various states of immunosuppression (Chen and Mellman, 2017). Angiogenesis, desmoplasia and immunosuppression are dysregulated in tumors, and hypoxia is a central downstream effect that leads to disease progression through various mechanisms (Jain, 2014; Noman et al., 2015).

### Normalization of the Tumor Microenvironment

Given the central role of hypoxia in poor outcome in patients with cancer and the dependence of oxygen delivery on blood vessels, the normalization hypothesis calls for increasing the function of vessels by modulating stromal cells towards a normal phenotype to enhance the efficacy of chemo-, radio-, and immunotherapies (Jain, 2001; Jain, 2014; Martin et al., 2019b). Though there are numerous physiological mechanisms that can be altered, the two critical abnormalities to be reversed to normalize blood vessels are leakiness and compression (Jain, 2014; Martin et al., 2019b).

**FIGURE 2:**
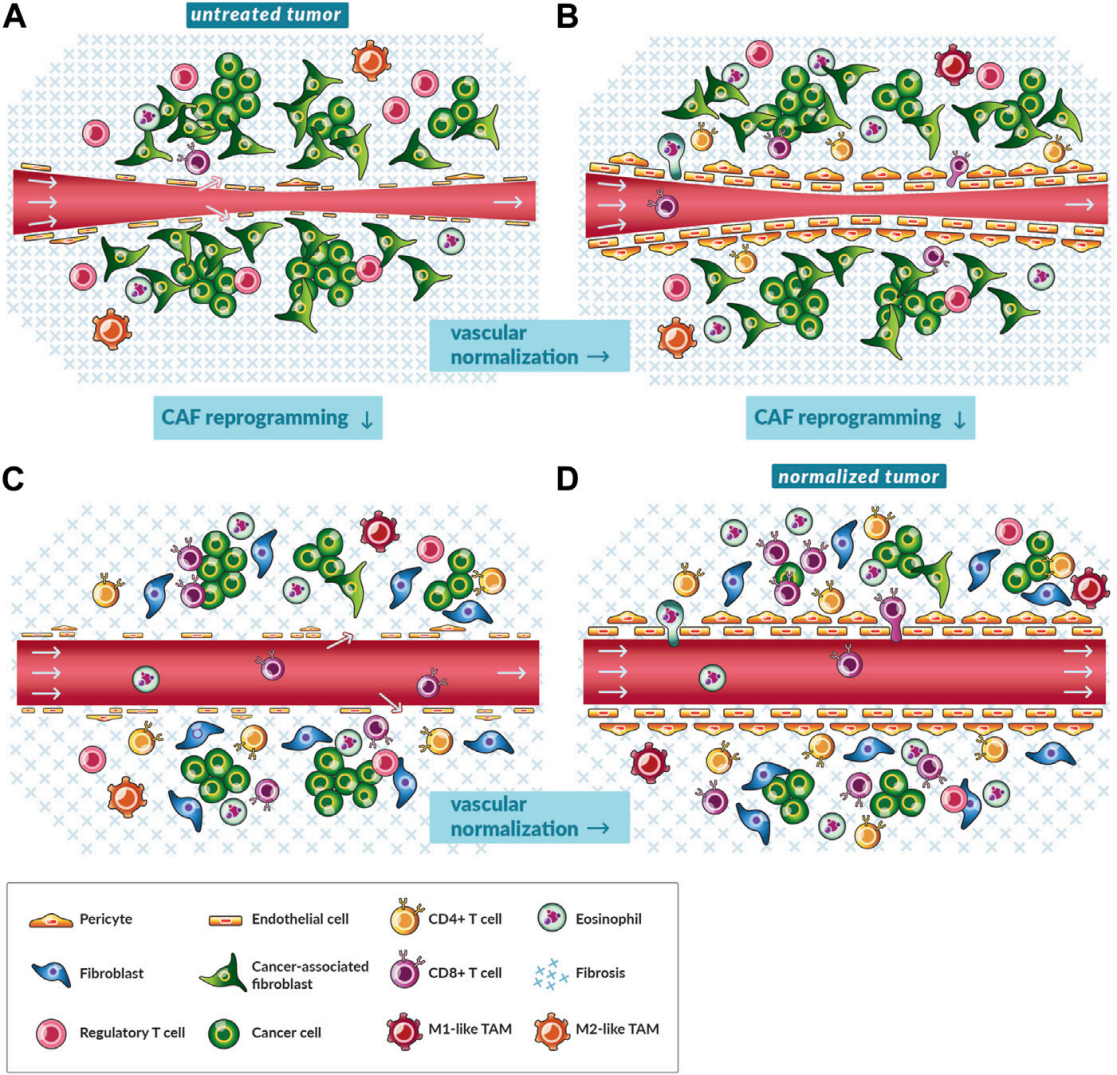
Vascular normalization and reprogramming of cancer-associated fibroblasts shift the microenvironment towards antitumor immunity. **(A)** A schematic of a magnified, cross-sectional view of a single blood vessel in an untreated tumor. A pinch point in the blood vessel (red tube) and the lack of a consistent endothelial cell layer fortified with pericytes restricts blood flow (gray arrows). The tumor is replete with immunosuppressive cancer-associated fibroblasts (CAFs), fibrosis, and regulatory T cells (CD4^+^CD25^+^FOXP3^+^) while lacking CD8^+^ and other subsets of CD4^+^ T cells. **(B)** A schematic of a magnified, cross-sectional view of a single blood vessel in a tumor treated with vascular normalizing therapy. As the balance of pro- and anti-angiogenic factors shifts towards the latter, endothelial cells are aligned, and blood vessels are fortified with pericytes yet remain compressed by mechanical stress. Perfusion increases especially in tumors with low levels of mechanical stress. Immune cells such as CD8^+^ T cells more efficiently traffic to tumors and transmigrate across the vessel wall. There are fewer immunosuppressive cells because of reduced angiogenic and hypoxia signaling. Vascular normalization by immune checkpoint inhibitors could rely on the accumulation of activated eosinophils. **(C)** A schematic of a magnified, cross-sectional view of a single blood vessel in tumor treated with CAF reprogramming therapy. As CAFs shift to quiescent fibroblasts, they produce and maintain lower levels of fibrosis. Mechanical stress is alleviated and vessels are decompressed. Perfusion increases. Immune cells such as CD8^+^ T cells flow through tumors and migrate the interstitial space because of less immunosuppressive CAF and hypoxia signaling. Also, there is less physical restriction of migration by components of fibrosis, such as collagen. **(D)** A schematic of a magnified, cross-sectional view of a single blood vessel in tumor treated with both vascular normalizing and CAF reprogramming therapy. Given the reduced signaling and physical barriers, immune cells such as CD8^+^ T cells efficiently traffic to tumors, negotiate transport through the vessel wall, and penetrate to clusters of cancer cells.

There are two types of TME normalization strategies. One alleviates blood vessel leakiness (vascular normalization, Figures 2A, B) and the other reverses compression (CAF reprogramming, Figures 2A, C). TME normalization usually refers to a therapeutic strategy to ‘normalize’ the balance of pro- and anti-factors of angiogenesis and/or desmoplasia signaling (Jain, 2014). In regards to angiogenesis, Jain introduced the hypothesis of vascular normalization to explain the paradox that, despite the requirement of angiogenesis for tumor growth, starving tumors of their blood supply by therapeutically inducing vascular regression did not improve patient outcome (Jain, 2001). Instead, preclinical studies demonstrated that balancing elevated pro-angiogenic signaling levels found in tumors with AATs will make the blood vessels phenotypically normal with increased fortification by perivascular cells and ECM (Figures 2A, B) (Tong et al., 2004; Winkler et al., 2004). As a result, the blood vessels function normally with decreased vessel leakiness, hypoxia and treatment resistance (Jain, 2014; Martin et al., 2019b). Blood vessel normalization has been extensively evaluated preclinically and in patients with cancer for combination with chemo-, radio-, and immunotherapies (Jain, 2014; Vasudev and Reynolds, 2014; Viallard and Larrivée, 2017; Martin et al., 2019b; Pinter et al., 2021), and vascular normalization could improve responses to ICIs through various mechanisms (Figures 2A, B) (Fukumura et al., 2018; Huang et al., 2018; Khan and Kerbel, 2018; Lee et al., 2020). Indeed, in patients with HCC, the combination of AAT and ICI, but not ICI monotherapy (Finn et al., 2019; Yau et al., 2019), outperforms AAT monotherapy (Finn et al., 2020).

The second type of normalization involves reversing vessel compression with CAF reprogramming therapies. Hereby, CAFs are turned quiescent such that they produce a smaller magnitude of forces and less amount of ECM such that there is a lesser magnitude of force generated and transmitted within tumors (Figures 2A, C) (Chauhan et al., 2013; Sherman et al., 2014; Incio et al., 2015; Whatcott et al., 2015; Papageorgis et al., 2017; Polydorou et al., 2017). Some CAF reprogramming therapies have been studied in preclinical, retrospective clinical, and prospective clinical studies. One such drug is losartan, which is an anti-hypertensive drug with decades of use in patients with high blood pressure. Losartan and other angiotensin system inhibitors reprogram CAFs to a quiescent phenotype through antagonism of the angiotensin II type I receptor (Chauhan et al., 2013). Dozens of retrospective studies indicated that patients with certain cancer types receiving angiotensin system inhibitors lived longer (Liu et al., 2017; Pinter and Jain, 2017; Zhao et al., 2019; Martin et al., 2020). Moreover, losartan improved the outcome of patients with pancreatic ductal adenocarcinoma undergoing chemoradiation in a prospective clinical trial (Murphy et al., 2019).

CAF reprogramming also appears to be combinable with ICI (Chauhan et al., 2019; Elahi-Gedwillo et al., 2019; Chen et al., 2019; Panagi et al., 2020; Mpekris et al., 2021; Voutouri et al., 2021). For instance, angiotensin system inhibitors prolonged survival of patients with certain tumor types undergoing ICI in a retrospective analysis (Drobni et al., 2022). Moreover, losartan had direct immunomodulatory effects on immune cells *in vitro* (Regan et al., 2019) and in clinical studies induced antitumor immunity (Liu et al., 2017) (Figures 2A, C). Additionally, losartan and other angiotensin signaling inhibiting drugs may ameliorate side effects of immunotherapy by enabling ICI dose reduction and inhibiting cytokine storm (Pinter et al., 2018). With this rationale, ICIs are now being tested prospectively with this combination of losartan and chemoradiation (NCT03563248). Like losartan, metformin is another drug with many effects in cancer and other diseases that has been repurposed to reprogram CAFs (Incio et al., 2015). There is evidence from the clinic that it modulates the TME towards antitumor immunity (Wang et al., 2020). Additionally, some drugs induce both vascular normalization and CAF reprogramming (Figure 2), such as the glucocorticoid steroid dexamethasone (Martin et al., 2019a), but the immunosuppressive properties of this drug are detrimental in many patients taking ICIs (Arbour et al., 2018). While vascular normalization and CAF reprogramming therapies have advanced to the clinic (Supplementary Tables 1, 2), they have yet to be tested in combination. Nonetheless, mathematical models and preclinical studies demonstrate the value of combining the normalization strategies for ICI (Figure 2D) (Mpekris et al., 2020; Panagi et al., 2020; Mpekris et al., 2021).

There are three important aspects to TME normalization. First, stromal cells in the tumor should be reprogrammed towards a non-diseased phenotype. Second, hypoxia should be alleviated, because TME normalization therapies can exacerbate hypoxia at high doses. Specifically, AAT administered at higher doses and for longer times prunes an excessive amount of blood vessels thereby reducing blood flow to the tumor (Martin et al., 2022). Besides inducing hypoxia, rapid depletion of stromal components including blood vessels (De Bock et al., 2011), pericytes (Cooke et al., 2012), CAFs (Rhim et al., 2014), hyaluronan (Wang-Gillam, 2019), collagen (Chen et al., 2021), and regulatory T cells (Zhang et al., 2020) through either genetic or pharmacological methods results in disease progression. Thus, the third aspect is that stromal components should reprogrammed rather than destroyed (Whatcott et al., 2015).

### Normalization by Immunotherapies

ICIs are approved for dozens of cancer types, but they only benefit a fraction of patients with cancer (Haslam and Prasad, 2019). To overcome primary resistance mediated by immunosuppressive hypoxia, angiogenesis and fibrosis signaling, researchers and oncologists are developing ICI combination therapy strategies including with vascular normalizing therapies (Fukumura et al., 2018), CAF reprogramming and nanomedicine (Martin et al., 2020; Martin and Jain, 2020). In addition to AAT and CAF reprogramming therapies, the contribution of ICI to TME normalization is under investigation currently (Mpekris et al., 2020). Thus, understanding the mechanisms through which immunotherapies normalize the TME could lead to more effective TME normalization and immunotherapy regimens.

There is both preclinical and clinical evidence that ICI monotherapy normalizes blood vessels in tumors that respond to ICI (Zheng et al., 2018). In murine breast tumors that respond to ICI with slowed tumor growth, ICI efficiently prunes vessels resulting in enhanced perfusion (Zheng et al., 2018). However, depletion of CD8^+^ T cells or inhibition of IFNγ production reversed the enhanced perfusion that ICI induced (Zheng et al., 2018). These results demonstrate that effective ICI therapy, which necessarily promotes CD8^+^ T cell accumulation and IFNγ production, also increases perfusion. Furthermore, these results generate the hypothesis that enhanced perfusion is a biomarker of response to ICI treatment (Zheng et al., 2018).

Both IFNγ and CD8^+^ T cells are necessary for antitumor immunity (Patel et al., 2017). At high levels, IFNγ induces apoptosis of cancer cells (Song et al., 2019). At low levels, IFNγ induces cancer cell stemness resulting in increased metastasis (Song et al., 2019). Also, IFNγ has non-immune-mediated antiangiogenic properties (Fathallah-Shaykh et al., 2000), and the extent of these effects could depend on the levels of IFNγ (Huang et al., 2018). AAT at high doses induce vascular regression while low doses induce vascular normalization (Jain, 2014; Martin et al., 2016). Similarly, at high levels of IFNγ in inducible models producing ∼10 ng per ml (Huang et al., 2018), IFNγ acts on stromal cells independently of cancer cells to induce vascular regression and eliminate blood flow to tumors (Kammertoens et al., 2017). At lower levels generated by adoptive cell transfer or ICI, IFNγ might induce vascular normalization, as it induces upregulation of ICAM-1 (Rothlein et al., 1988), which promotes adhesion between endothelial cells and leukocytes, and VCAM-1 (Wang et al., 2007), which promotes adhesion between pairs of endothelial cells and endothelial cells and leukocytes or mural cells. Separately, through IFNγ, CD8^+^ T cells can polarize TAMs to an M1-like phenotype, which is antiangiogenic (De Palma et al., 2017). ICI also depends on IFNγ to induce antitumor immune responses (Patel et al., 2017). ICI, TAM polarization, adoptive cell transfer and experimental models of inducible IFNγ all introduce different concentrations of IFNγ in the TME, thereby, inducing different magnitudes of antiangiogenic effects (Huang et al., 2018). Thus, vascular normalization resulting from immunotherapies depends on the context and requires further study.

In patients with glioblastoma, tumors responding to ICI had reduced vascular permeability indicative of vascular normalization (Qin et al., 2017). However, this reduction occurred 6 months after ICI treatment initiation and after a brief period of increased vascular permeability. Therefore, the conflicting kinetics of these processes between murine breast tumors and glioblastoma tumors in patients must be resolved by observing vascular permeability in tumors from patients and related murine models at times before and shortly after ICI treatment initiation (De Palma and Jain, 2017). Additionally, vascular regression associated with ischemic tumor necrosis after vaccination and/or ICI was observed in responding melanoma and ovarian tumors from patients (Schoenfeld et al., 2010). Thus, the kinetics and tumor type dependence of the antiangiogenic effects of immunotherapies in tumors in patients must be clarified.

The cells responsible for producing the IFNγ that modulates the vasculature differ between studies. In experiments resembling the clinical ‘preventative setting’ in which interventions occur before tumors could be diagnosed, knockout models revealed that CD8^+^ T cells and CD4^+^ T cells had opposite angiogenic effects. CD8^+^ T cells induced endothelial cell proliferation, yielding a pro-angiogenic effect. In contrast, the CD4^+^ T cells induced pericyte recruitment, a critical process for vascular normalization and alleviation of hypoxia (Tian et al., 2017). Thus, in the ‘preventative setting’, CD4^+^ T cells through IFNγ seem to be responsible for vessel fortification through pericyte recruitment (De Palma and Jain, 2017; Tian et al., 2017) consistent with previous studies of angiogenesis in early tumor development (Beatty and Paterson, 2001).

**FIGURE 3:**
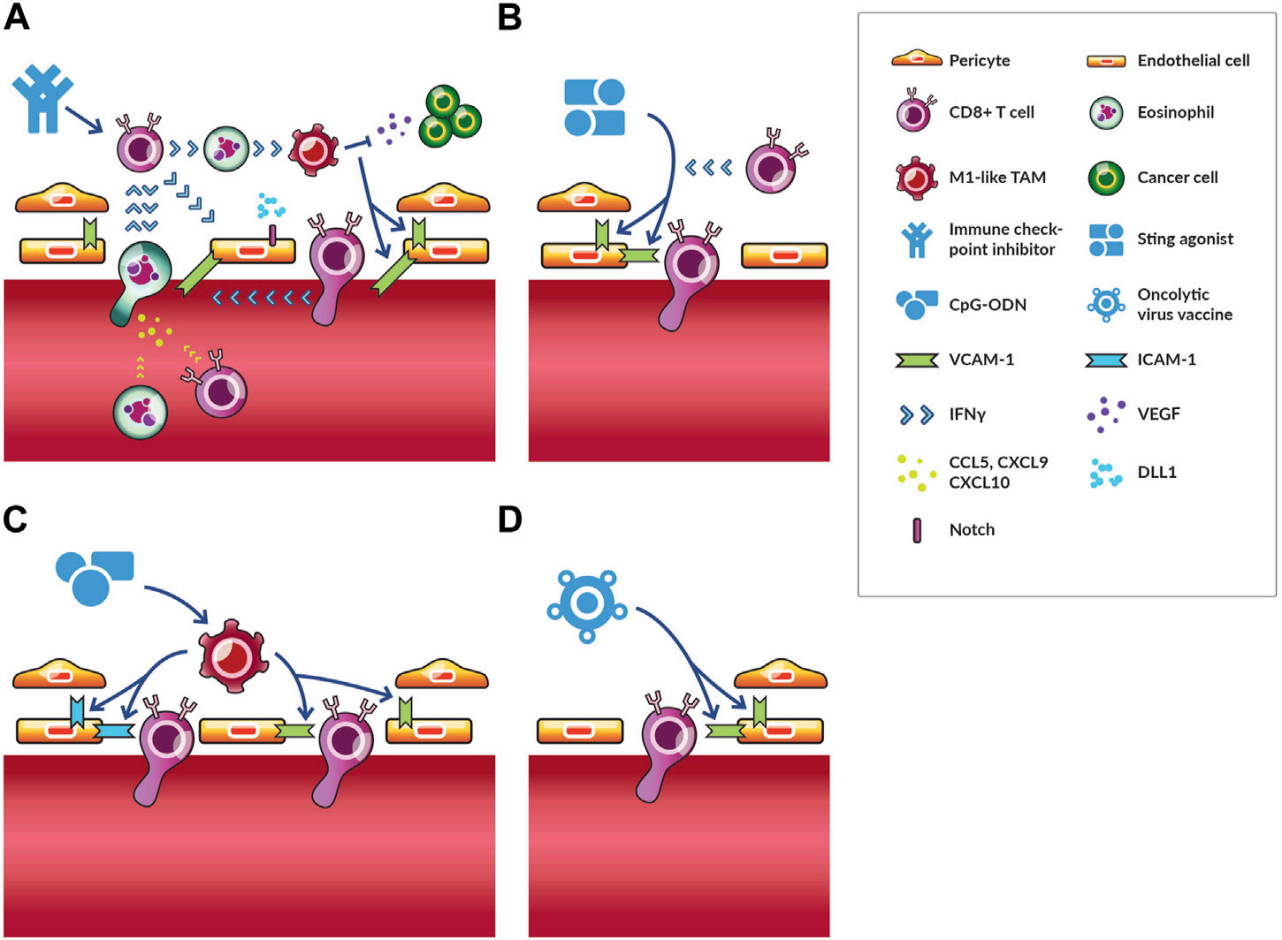
Vascular normalization through immunotherapies. Immunotherapies stimulate various immune cells to act on endothelial and mural cells resulting in vascular normalization. Several immunotherapies normalize vessels through distinct mechanisms. **(A)** The effects of immune checkpoint inhibitors (ICIs) are the most well-studied. ICIs activate CD8^+^ T cells, which secrete IFNγ. These cells can interact with activated eosinophils through IFNγ to induce M1-like TAM phenotypes, which reduces VEGF signaling and induces VCAM-1 expression. As a result, more CD8^+^ T cells and activated eosinophils adhere to and transmigrate across the endothelium. The later secretes chemokines (i.e., CCL5, CXCL9 and CXCL10), which increase trafficking of CD8^+^ T cells and eosinophils to tumors. For this reason, activated eosinophil accumulation precedes and is required for increased CD8^+^ T cell homing to tumors. This process is a potential feedback loop of vascular normalization and antitumor immunity. DLL1-Notch signaling promotes CD8^+^ T cell activation and IFNγ production thereby reinforcing this positive feedback loop for long-term vascular normalization. **(B)** STING agonists cause an increase in antiangiogenic factors, which results in increased pericyte coverage through VCAM-1 expression, which also facilitates the infiltration of T cells. **(C)** CpG-ODN directly act on TAMs promoting an M1-like phenotype, which induces the upregulation of ICAM-1 and VCAM-1 expression. In some contexts, depletion of regulatory T cells has similar effects on ICAM-1 and VCAM-1 expression. **(D)** Oncolytic vaccines reduce vascular density and increase VCAM-1 expression through unelucidated mechanisms.

Interventions with ICI in the time window when treatment would typically occur in patients normalized the tumor vasculature in an IFNγ-dependent manner, with lack of effect using IFNγ receptor knock out mice and upon IFNγ-neutralization studies (Zheng et al., 2018). In T cell depletion studies in wild type mice, ICI-induced normalization and enhanced perfusion proved to be dependent on CD8^+^ but not CD4^+^ T cells in murine breast tumors (Zheng et al., 2018). Taken together with the results from the preventative setting, these studies collectively indicate that CD4^+^ T cells play a role in vessel maturation early in tumor development while ICIs act through CD8^+^ T cells to normalize blood vessels in the treatment setting. The notion that immunotherapy-induced vascular normalization is mediated by CD8^+^ T cells in tumors in the treatment setting is supported by observations that adoptive transfer therapy of T cells contributes to normalized vascular morphology (Ganss et al., 2002). Interestingly, such ICI-induced normalization also depended on eosinophil accumulation in murine breast tumors in addition to CD8^+^ T cell accumulation and IFNγ (Zheng et al., 2020). These findings are consistent with previous studies describing crosstalk between eosinophils and CD8^+^ T cells that leads to a positive feedback loop of vascular normalization and antitumor immunity (Carretero et al., 2015). Furthermore, activated eosinophils in the TME, through IFNγ, skew TAMs to an antiangiogenic M1-like phenotype. As a result, there is some vascular normalization with elevated expression of VCAM-1, which induces adhesion of eosinophils and T lymphocytes (among other cells) to the endothelium thereby promoting transmigration and infiltration. In turn, these T cells can promote more vascular normalization and skewing of TAMs. Additional tumor-specific effector T cells continue to be trafficked to the tumor by interferon-induced chemoattractants produced by activated eosinophils (Carretero et al., 2015). In patients, increases in eosinophil and lymphocyte counts after ICI correlated with increased survival (Delyon et al., 2013). Thus, during ICI treatment there is a positive feedback loop of CD8^+^ T cells and activated eosinophils stimulating antiangiogenic effects directly through IFNγ production and indirectly through TAM polarization, which in turn increases eosinophil and lymphocyte adhesion to the endothelium and tumor accumulation (Figure 3A).

Another observation of long-term vascular normalization and antitumor immunity by activated CD8^+^ T cells, though not an example of immunotherapy causing vascular normalization, adds further support to this positive feedback cycle hypothesis (Zhang et al., 2021). Specifically, researchers studied the ligand Delta-like canonical Notch ligand 1 (DLL1) (Huang et al., 2011). The levels of this ligand are reduced with increased circulating VEGF, which is characteristic of tumors with high levels of angiogenesis (Huang et al., 2011). When DLL1-Notch signaling is reduced in bone marrow precursors by circulating VEGF, T cell activation and antitumor immunity is reduced (Huang et al., 2011). However, interfering with circulating VEGF-induced DLL1-Notch signaling inhibition by overexpressing DLL1 in cancer cells induced long-term vascular normalization (Zhang et al., 2021). This normalization was dependent on IFNγ production and CD8^+^ T cell accumulation (Zhang et al., 2021). In turn, this long-term vascular normalization was necessary for ICI efficacy in the resistant tumor model assayed (Zhang et al., 2021). Thus, long-term vascular normalization can be induced by immune cells through interference of angiogenic and immunosuppressive signaling, and this normalization can increase the antitumor effect of ICI (Figure 3A).

Besides ICI, other immunotherapies have normalizing effects. In melanoma, low-dose local administration of a STING agonist increased expression of antiangiogenic factors resulting in increased endothelial cell pericyte coverage and expression of VCAM-1 (Chelvanambi et al., 2021). In this case, vascular normalization depended on STING activation of dendritic cells rather than effects on cancer cells, which also express STING receptor (Chelvanambi et al., 2021). These normalized vessels, along with newly formed tertiary lymphoid structures, increased T cell infiltration (Chelvanambi et al., 2021). These processes were studied in more detail in breast, lung, and colorectal cancer models (Yang et al., 2019). As in the study in melanoma, STING agonism induced expression of vascular stabilization genes, but there were several additional findings important to understanding STING agonist-induced vascular normalization. First, while STING agonism of hematopoietic stromal cells like dendritic cells were necessary for immune response, agonism of nonhematopoietic stromal cells, particularly endothelial cells, mediated the normalization process (Yang et al., 2019). Second, STING agonist-induced vascular normalization depended on CD8^+^ T cells and IFNγ but not TAMs (Yang et al., 2019). Taken together, these results demonstrate that STING agonists, like ICI, can induce vascular normalization through CD8^+^ T cells and IFNγ leading to enhanced T cell vascular adhesion, infiltration, and therapeutic effects (Figure 3B).

Other immunostimulatory agents, such as oligodeoxynucleotides (ODN) with cytosine-guanine-rich (CpG) motifs (CpG-ODN) normalize vessels as evidenced by the upregulation of ICAM-1 and VCAM-1 in endothelial cells by TAMs directly stimulated by CpG-ODN (Garbi et al., 2004). With such normalized vessels, adoptively transferred immune cells could better extravasate and infiltrate tumors (Figure 3C). Despite clinical testing, CpG-ODN has not succeeded, perhaps in part of the necessity of local administration. Even if CpG-ODN is effective in the tumor in which it is administered and the patient develops systemic antitumor immunity through an abscopal effect, the TME of metastatic lesions could still impair infiltration (Martin et al., 2020). Similarly to the effects of CpG-ODN, in some contexts depletion of regulatory T cells also increases the upregulation of ICAM-1 and VCAM-1 adhesion molecules (Li et al., 2010). Depletion of regulatory T cells also lead to other indicators of vascular normalization including reduced vessel diameter and increased perfusion (Li et al., 2010). Combinations of antiangiogenic therapy and ICI could promote infiltration and activation, respectively, of regulatory T cells, so depletion of regulatory T cells through anti-TAM therapy and chemotherapy can be beneficial (Martinez-Usatorre et al., 2021). While depletion of regulatory T cells generates antitumor immunity in most contexts, researchers reported that depletion of regulatory T cells depletes pancreatic ductal adenocarcinoma tumors of fibroblasts, which paradoxically unleashes tumor growth and immunosuppression (Zhang et al., 2020). Thus, the effect of regulatory T cell depletion could depend on the tumor type. Finally, oncolytic viruses can also reduce vascular density transiently while increasing VCAM-1 gene expression (Chon et al., 2019), but through what mechanisms and whether vessels are normalized versus regressed is unclear, as is whether hypoxia is alleviated (Figure 3D). Thus, whereas STING agonists seem to act through T cells and IFNγ as with ICI treatment, oligonucleotide therapies might act through TAMs to increase expression of adhesion molecules. Further research is necessary to determine whether and how these immunotherapies are combinable for enhanced vascular normalization

### Combination Therapies for Tumor Microenvironment Normalization

Several rationales have been developed for combining AATs and ICI with disparate effects on vascular regression and normalization. One mechanism of synergy involves AATs inducing vascular changes resulting in more recruitment of CD8^+^ T cells. Although this mechanism is resisted by IFNγ acting on endothelial cells to upregulate immune checkpoint expression, this resistance can be neutralized through ICI (Schmittnaegel et al., 2017). This mechanism was investigated in the context of combined VEGF and angiopoietin 2 inhibition, which induced vascular regression leading to tumor necrosis (Schmittnaegel et al., 2017). The remaining vessels were normalized as evidenced by increased pericyte coverage, but the density of vessels was low. This ‘passive vessel normalization’, in which immature blood vessels are destroyed while the mature vessels remained, was sufficient to facilitate recruitment of CD8^+^ T cells. Thus, there are mechanisms of synergy between AATs and ICI that are effective when active vascular normalization through pericyte recruitment to immature vessels does not occur while vascular regression does occur. A separate study demonstrated that VEGF inhibition and ICI were complimentary through formation of high endothelial venules, which are blood vessels critical for the recruitment of T cells to antigen-presenting cells within tertiary lymphoid structures or lymph nodes, leading to increased lymphocyte infiltration (Allen et al., 2017). As in the other study, researchers observed regression of half of the tumor vasculature (Allen et al., 2017). A unique aspect was the investigation of the independent contributions of AAT and ICI to vessel pruning and fortification. While anti-VEGF therapy induced vascular regression and fortification, the addition of ICI did not contribute to vascular regression yet further increased pericyte coverage (Allen et al., 2017). Thus, ICI induced active pericyte recruitment, which occurred through an increase in the angiostatic properties of myeloid cells in the TME (Allen et al., 2017). After tumor relapse from AAT monotherapy, only the combination of AAT and ICI could reduce vessel density, suggesting the antiangiogenic properties of ICI were non-redundant with VEGF inhibition (Allen et al., 2017). Thus, there are mechanisms of synergy between AAT and ICI that induce vascular regression and are independent of vessel normalization, but ICI seems to have stronger effects on vessel fortification rather than regression.

**FIGURE 4:**
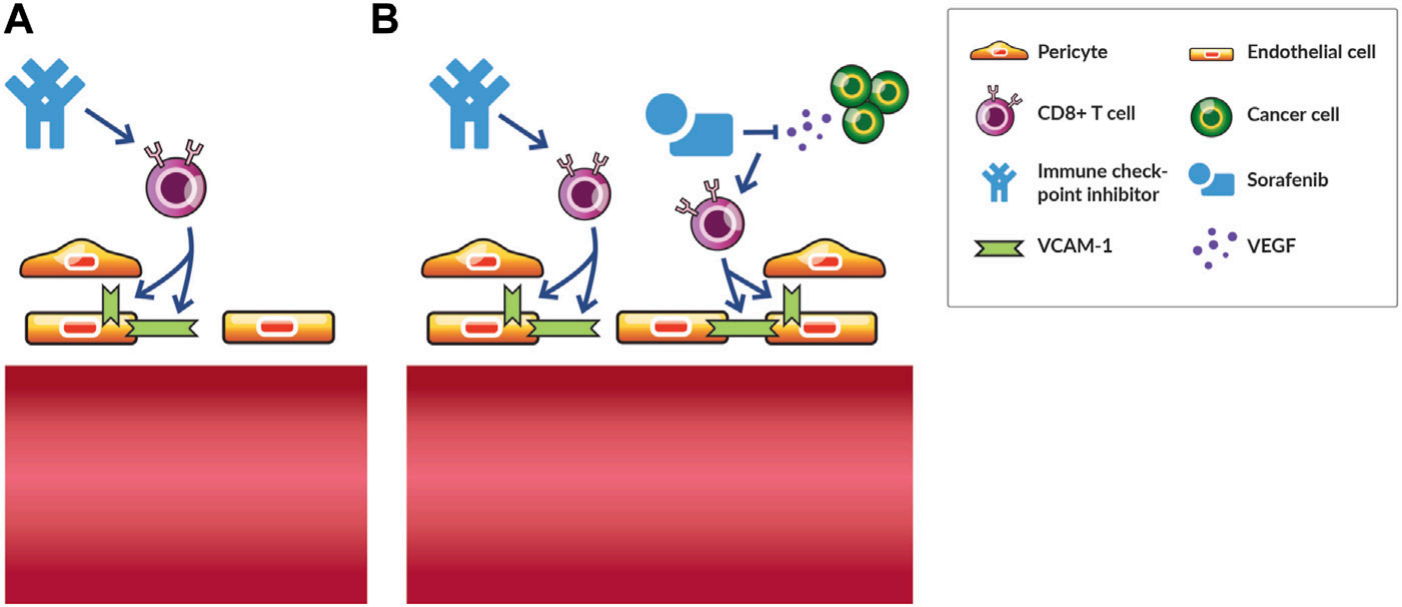
Vascular fortification by immune checkpoint inhibition promotes vascular normalization by subsequent antiangiogenic therapy in hepatocellular carcinoma. **(A)** Immune checkpoint inhibition monotherapy normalizes tumor blood vessels in various murine models of cancer by increasing the interaction between endothelial cells and with perivascular cells in a CD8^+^ T cell dependent manner. **(B)** Following immune checkpoint inhibition with antiangiogenic therapy, such as the small-molecule tyrosine kinase inhibitor sorafenib, increases the density of tumor blood vessels fortified by pericytes and antitumor efficacy in a CD8^+^ T cell dependent manner. In contrast, sorafenib monotherapy induces vascular regression and does not induce an antitumor effect. Thus, vascular-fortifying ICI can shift the effect of subsequent antiangiogenic therapy from vessel destruction to normalization.

A series of reports in HCC provided an illustrative case of combined AAT and ICI vascular normalization. Sorafenib, which was the only approved AAT for HCC at the time, excessively pruned vessels, thereby, causing hypoxia and stimulating the SDF1α/CXCR4 pathway (Chen et al., 2015). Although blocking CXCR4 reduces fibrosis and increases T cell infiltration in some desmoplastic tumor types (Chen et al., 2019), in highly vascular HCC after relapse from AAT, blocking CXCR4 alleviated immunosuppressive cell recruitment and angiogenesis resistance mechanisms to some extent (Chen et al., 2015). However, adding ICI to AAT and CXCR4 inhibition alleviated these resistance mechanisms further by increasing the amount of IFNγ and CD8^+^ T cells in the tumor center, indicating that vessels could be further normalized (Chen et al., 2015). Indeed, a follow-up study demonstrated that ICI induced vessel normalization to a larger extent through CD4^+^ T cells when combined with an anti-VEGFR2 antibody compared to the AAT antibody alone (Shigeta et al., 2019). Anti-VEGFR2 antibody increased the endothelial cell expression of PD-L1 in an IFNγ-dependent manner and PD-1 expression in CD4^+^ T cells providing further rationale for combination therapy with ICI (Shigeta et al., 2019). Importantly, ICI-induced fortification of vessels (Figure 4A) prevented vascular regression caused by higher doses of anti-VEGFR2 antibody thereby increasing the therapeutic index of this AAT (Shigeta et al., 2019). This finding is important given the sensitivity of vascular normalization to anti-VEGF therapy dose (Jain, 2014). Interestingly, when the AAT sorafenib follows ICI in HCC, the density of blood vessels associated with pericytes increases in a CD8^+^ T cell dependent manner (Figure 4B), while sorafenib not preceded by ICI causes vascular regression (Kikuchi et al., 2022). Thus, ICI fortifies vessels thereby preventing vascular regression induced by subsequent AAT. Along these lines, administering AAT to normalize vessels avoided oncolytic vaccine therapy-induced vascular regression and improved therapeutic efficacy (Matuszewska et al., 2019). Thus, combinations of AAT and ICI can be administered in various schedules to induce a greater extent of vascular normalization while avoiding regression.

While in certain contexts the combination of ICI and AAT prevents vascular regression caused by high levels of IFNγ or high doses of AAT, respectively, in other contexts vascular regression is dependent on the dose of AAT. Specifically, high doses of the small-molecule tyrosine kinase VEGF inhibitor regorafenib combined with ICI in HCC induced vascular regression (Shigeta et al., 2020). Besides dose-dependent normalization, the authors demonstrated that regorafenib induced CXCL10 expression in HCC cells. This chemokine binds to its receptor CXCR3 expressed on circulating lymphocytes to increase their trafficking to tumors (Shigeta et al., 2020). This study provides an additional hypothesis for a feedback loop between T cells and vascular normalization. Specifically, regorafenib and ICI normalize the vasculature and increase CXCL10 expression resulting in increased infiltration of T cells, which induce additional vascular normalization. One AAT resistance mechanism associated with gene expression changes, albeit in stromal cells in this case, involves upregulation of the EGFR pathway in endothelial and perivascular cells of lung adenocarcinoma (Cascone et al., 2011). Accordingly, blocking EGFR is effective for treating VEGF-resistant tumors and combining EGFR inhibition with immunotherapies might result in TME normalization. EGFR inhibition using fusion proteins or bispecific antibodies also targeting death receptors (Bremer et al., 2005), immune checkpoints (Koopmans et al., 2018), or CD47 (Hendriks et al., 2020) signaling have also been developed and their vascular normalization properties should be evaluated.

There is also preclinical evidence that other immunotherapies besides ICI, when combined with AATs, reinforce vascular normalization and induce CAF reprogramming. Specifically, an anti-CD40 antibody, which promotes dendritic cell maturation, antigen presentation and priming of T cells, demonstrated normalization properties. In genetically engineered colorectal cancer models, researchers demonstrated that adding anti-CD40 antibody to combined anti-VEGF and anti-angiopoietin two therapy fortified blood vessels even in non-angiogenic tumors (Ragusa et al., 2020). Interestingly, in this study AAT alone induced CAF reprogramming effects, and this effect was increased with anti-CD40 antibody (Ragusa et al., 2020). Of note, TME normalization was T cell independent, even though the antitumor efficacy depended on T cells (Ragusa et al., 2020). Nonetheless, the antitumor activity was also dependent on angiogenic (*i.e.*, angiopoietin 2) signaling (Ragusa et al., 2020). An interesting aspect of this study is that the tumors were well-perfused at baseline, so the increases observed in antitumor activity appear to be from increased trafficking and infiltration of T cells to tumors resulting from increased vessel maturity and increased T cell migration resulting from reduced fibrosis (Ragusa et al., 2020). Unlike in other studies, this study was performed in well-perfused tumors and normalization did not depend on T cells, so combination of anti-CD40 and ICI therapies, such as through fusion proteins (Medler et al., 2022) and bispecific antibodies (Salomon et al., 2022), might normalize vessels through non-redundant mechanisms. Overall, these studies demonstrate that immunotherapies enhance vascular normalization and CAF reprogramming when combined with AAT, and the resulting increase in antitumor efficacy can occur through various mechanisms that can be independent of increased perfusion and alleviated hypoxia.

## Discussion

TME normalization, which reduces hypoxia, is a potentially promising approach to enhance responses to immunotherapy based on the preclinical body of evidence, but clinical data remains to be generated. Immune cells modulate tumor vasculature. Accordingly, various immunotherapies including ICIs, oncolytic viral vaccines, and immunostimulatory therapies such as STING agonists induce antiangiogenesis, often through IFNγ and CD8^+^ T cells, but also through promoting angiostatic properties in TAMs. The tumor type and amount of IFNγ produced seem to determine the extent of antiangiogenic effect, with large amounts of IFNγ leading to vascular regression and hypoxia rather than normalization and normoxia. In preclinical cancer models, efficacious ICI stimulates CD8^+^ T cells and IFNγ production thereby fortifying vessels with pericytes leading to increased perfusion. This process could stimulate a positive feedback loop of increased T cell recruitment and normalization. Further work could clarify how different immune cells differentially regulate aspects of vascular normalization, including vessel pruning and pericyte recruitment. Additionally, the tumor type dependence and kinetics of vascular normalization in patients must be studied further.

AAT and ICI combinations are efficacious through various mechanisms and can induce vascular regression or normalization (Supplementary Table S3). In either case, ICI contributes to the fortification of blood vessels with pericytes and combination with AAT increases immune cell recruitment. Fortifying vessels with immunotherapies can help avoid excessive vessel pruning by high doses of AAT. Alternatively, normalizing vessels with AAT can inhibit vascular regression caused by subsequent immunotherapy. These effects seem to depend on the tumor type, treatment type, and kinetics of response. By understanding these interactions, long-lasting normalization might be more effectively achieved by combination immunotherapies with AATs. To what extent CAF reprogramming therapy through alleviation of hypoxia and immunosuppressive signaling can increase the efficacy of combined AAT and ICI remains unclear. Relatedly, further research should test whether immunotherapies reprogram CAFs. The more effectively the positive feedback loop of activated immune cells inducing vascular normalization can be harnessed, the more effectively immunotherapies can induce antitumor immunity.
