# Supplementary material for: Towards Immunotherapy-Induced Normalization of the Tumor Microenvironment
Source: Front Cell Dev Biol. 2022 May 30;10:908389. doi: 10.3389/fcell.2022.908389 (PMC9196132; doi:10.3389/fcell.2022.908389)
Supplement: Supplementary file 1 [file DataSheet1.docx]

**Supplementary information**

**Towards immunotherapy-induced normalization of the tumor microenvironment**

Vinicio Melo^1^, Edwin Bremer^1^, John D. Martin^2,*^

**Author affiliations**

^1^Department of Hematology, University of Groningen, University Medical Center Groningen, Groningen, Netherlands

^2^Materia Therapeutics, Las Vegas, NV, USA.

**Keywords:**

tumor microenvironment, vascular normalization, immune checkpoints, hypoxia, immunotherapy, angiogenesis, immune cell infiltrate

**Running title:**

Immunotherapy-Induced Tumor Microenvironment Normalization

*Correspondence to:

[jdmartin@alum.mit.edu](mailto:jdmartin@alum.mit.edu) (JDM)

**Supplementary Table 1. FDA-approved vascular normalization agents in clinical trials with immunotherapy.** Colors indicate FDA-approval, phase III clinical trial active or recruiting or not yet recruiting and no phase III clinical trial. Updated from the senior author’s previous work (1).

| Drug | Target | Approved indications | | Clinical trials in combination with ICBs | Brief Description |
| --- | --- | --- | --- | --- | --- |
| Axitinib | TKI (VEGFR-1–3, PDGFR-β) | RCC | Approved for: first line treatment of patients with advanced RCC with either avelumab or pembrolizumab  NCT02684006  RCC  NCT02853331  RCC | | Tested as a first line therapy in combination with avelumab in patients with RCC. |
| Bevacizumab | Anti-VEGF-A antibody | CRC, NSCLC, breast, RCC | Approved for: first line treatment of patients with metastatic non-squamous, NSCLC in combination with atezolizumab and chemotherapy.  Approved for treatment of patients with persistent, recurrent or metastatic cervical cancer whose tumors express PD-L1 (CPS ≥1) in combination with pembrolizumab plus chemotherapy.  Approved for treatment of patients with HCC in combination with atezolizumab  NCT03038100  Ovarian cancer  NCT04194203  NSCLC  NCT04102098  HCC  NCT04487067  HCC  NCT03737643  Advanced ovarian cancer  NCT02891824  Epithelial ovarian cancer  NCT03991403  NSCL  NCT03353831  Recurrent ovarian carcinoma  NCT03847428  HCC  NCT04712643  HCC  NCT04803994  HCC  NCT04732286  HCC  NCT05116189  Recurrent ovarian cancer  NCT04732598  Breast cancer  NCT03762018  Malignant pleural mesothelioma  NCT03778957  HCC  NCT03635567  Cervical cancer  NCT03740165  Ovarian cancer  NCT02997228  Metastatic colorectal adenocarcinoma  NCT02839707  Ovarian, fallopian tube, or primary peritoneal cancer  NCT03414983  CRC  NCT03556839  Cervical cancer  NCT03434379  HCC  NCT05063552  Head and neck cancer  NCT03178552  NSCLC | | Tested as first line therapy in combination with atezolizumab and chemotherapy in patients with NSCLC, HCC, malignant pleural mesothelioma, metastatic CRC, cervical cancer, and head and neck cancer.  Tested as adjuvant or first line therapy in combination with atezolizumab in patients with high risk of recurrence of HCC, unresectable HCC, unresectable HER2^-^ breast cancer, and metastatic HCC.  Tested as first line therapy in combination with durvalumab and chemotherapy in patients with advanced ovarian cancer.  Tested in combination with durvalumab as first line therapy in patients with advanced HCC.  Tested as a first line therapy in combination with transarterial chemoembolization and atezolizumab or durvalumab in patients with HCC unsuitable for curative therapy.  Tested as first line therapy in combination with pembrolizumab and chemotherapy in patients with persistent, recurrent, and metastatic cervical cancer.  Tested in combination with pembrolizumab as a first line therapy for recurrent ovarian cancer and NSCLC.  Tested as first line therapy in combination with nivolumab and chemotherapy in metastatic CRC. |
| Cabozantinib | TKI (VEGFR-2, Tie2) | Medullary thyroid, RCC, HCC | Approved for: combination with nivolumab as first-line treatment for patients with advanced RCC  NCT04338269  RCC  NCT03755791  HCC  NCT04471428  NSCLC  NCT03937219  RCC  NCT04446117  Prostate cancer  NCT05092958  Urothelial cancer  NCT03793166  Advanced kidney cancer | | Tested as a second line therapy in combination with atezolizumab patients with inoperable RCC, metastatic castration-resistant prostate cancer and metastatic NSCLC.  Tested as a first line therapy in combination with atezolizumab in patients with advanced HCC.  Tested as a first line therapy in combination with nivolumab and ipilimumab in patients with metastatic urothelial cancer.  Tested in combination with nivolumab following treatment with ipilimumab and nivolumab in patients with advanced kidney cancer. |
| Everolimus | mTOR inhibitor | RCC, PNET, GI, lung | None | |  |
| Thalidomide | Chemotherapy pleiotropic | Multiple myeloma | NCT02726581  Multiple myeloma | | Tested as a first line therapy in combination with nivolumab in patients with multiple myeloma. |
| Lenvatinib | TKI (VEGFR-1–3, PDGFR-α, FGFR1–4, KIT, RET) | DTC, RCC, HCC, endometrial carcinoma | Approved for: first line treatment of patients with renal cell carcinoma and endometrial carcinoma with pembrolizumab  NCT03713593  HCC  NCT04889118  Melanoma  [NCT03820986](https://clinicaltrials.gov/ct2/show/NCT03820986)  Melanoma  NCT03976375  NSCLC  NCT04776148  CRC  NCT03517449  Endometrial carcinoma  NCT03884101  Endometrial carcinoma  [NCT04676412](https://clinicaltrials.gov/ct2/show/NCT04676412)  NSCLC  [NCT03829332](https://clinicaltrials.gov/ct2/show/NCT03829332)  NSCLC  NCT04716933  NSCLC  NCT03829319  NSCLC  NCT02811861  RCC  NCT04770896  HCC | | Tested as a first line therapy in combination with pembrolizumab in patients with HCC and melanoma.  Tested in combination with pembrolizumab in patients with CRC, endometrial carcinoma, and metastatic NSCLC.  Tested as a first line therapy in combination with chemotherapy and pembrolizumab in patients with NSCLC.  Tested as a first-line therapy in combination with pembrolizumab in patients with advanced RCC.  Tested as a second line therapy in combination with atezolizumab in patients with locally advanced or metastatic HCC. |
| Nintedanib | TKI (VEGFR-2, PDGFR-α/β, FGFR-1) | NSCLC | None | |  |
| Pazopanib | TKI (VEGFR-1–3, PDGFR-β, FGFR-1–2) | RCC, soft tissue sarcoma | None | |  |
| Ramucirumab | Anti-VEGFR2 antibody | Gastric, HCC, NSCLC, CRC | None | |  |
| Regorafenib | TKI (VEGFR-1–3, PDGFR-β, FGFR-1–2) | CRC, GIST, HCC | NCT04879368  Gastro-oesophageal cancer | | Tested as a second line therapy in combination with nivolumab in patients with gastro-oesophageal cancer. |
| Sorafenib | TKI (VEGFR-2 & 3, KIT, Raf, PDGFR-β) | RCC, HCC, thyroid cancer | NCT04770896  HCC | | Tested as a second line therapy in combination with atezolizumab in patients with locally advanced or metastatic HCC. |
| Sunitinib | TKI (VEGFR-1–2, PDGFR-α/β, KIT, RET, CSFR-1, FLT3) | RCC, GIST, PNET | None | |  |
| Vandetanib | TKI (VEGFR-2) | Medullary thyroid cancer | None | |  |
| Aflibercept | Protein blocking VEGF (VEGF-A, VEGF-B, PlGF) | Metastic CRC | None | |  |
| Pralsetinib | TKI (RET, DDR1, TRKC, FLT3, JAK1-2, TRKA, VEGFR2, PDGFR-β, FGFR1-2) | NSCLC, advanced or metastatic RET-mutant and RET fusion-positive thyroid cancers, medullary thyroid cancer | None | |  |
| Infigratinib | FGFR 1-4 | Cholangiocarcinoma | None | |  |
| Tivozanib | TKI (VEGFR-1–3, PDGFR-β, KIT) | RCC | NCT04987203  RCC | | Tested in combination with nivolumab in patients with advanced RCC who have had 1 or 2 prior lines of therapy, one of which was an ICI. |
| Erdafitinib | FGFR 1-4 | Metastatic bladder cancer | None | |  |
| Pemigatinib | FGFR 1-3 | Cholangiocarcinoma | None | |  |
| Selpercatinib | TKI (RET) | Lung and thyroid cancers | None | |  |
| Ripretinib | **PDGFR-α/β** | GIST | None | |  |
| Pexidartinib | CSF1R, CD117, KIT, FLT3, PDGFR-β | TGCT | None | |  |
| Sirolimus | mTOR inhibitor | Perivascular epithelioid cell tumor | None | |  |
| Temsirolimus | mTOR inhibitor | RCC | None | |  |

On April 22, 2022 we searched clinicaltrials.gov for the [name of anti-angiogenic therapy] AND pembrolizumab OR atezolizumab OR nivolumab OR cemiplimab OR ipilimumab OR durvalumab OR avelumab OR ipilimumab AND Cancer [DISEASE] with filters for Phase III and active or recruiting.

**Supplementary Table 2. Investigational CAF reprogramming and extracellular matrix modifying agents in clinical trials with immunotherapy.** Colors indicate approval or clinical trial active or recruiting or not yet recruiting and no approval or no clinical trial. Updated from the senior author’s previous work (1).

| Drug name/class, target | FDA approved for cancer/other | Clinical stage with immunotherapy | |
| --- | --- | --- | --- |
| Losartan/angiotensin system inhibitors, TGF-β | No/yes | NCT03563248  Phase II ongoing in combination with nivolumab, radiation and FOLFIRINOX in PDAC | |
| Paricalcitol/vitamin D receptor agonist, vitamin D receptor | No/yes | NCT02930902  Phase II ongoing in combination with pembrolizumab and chemotherapy in resectable (NCT02930902, NCT03519308) or metastatic (NCT02754726)  PDAC) | |
| Plerixafor/immunostimulant, CXCL12/CXCR4 | Yes/yes | Phase II ongoing – in combination with cemiplimab in metastatic pancreatic cancer |  |
| Metformin/treatment for diabetes, TGF-β | No/yes | Phase II ongoing in combination with nivolumab in NSCLC (NCT03048500) and CRC (NCT03800602), pembrolizumab in melanoma (NCT03311308), and in metastatic head and neck (NCT04414540 and NCT04114136), advanced melanoma, RCC, NSCLC, HCC (Child Pugh Class A only), MSI-High solid tumors, urothelial cancer, GE junction/gastric adenocarcinoma, durvalumab in head and neck cancer (NCT03618654), and in breast cancer (NCT01042379) | |
| All-trans retinoic acid | Yes/yes | Phase II ongoing in melanoma in combination with pembrolizumab (NCT03200847) and ipilimumab (NCT02403778) | |
| PEGPH20/hyaluronidase, hyaluronan* | No/no | Early Phase I with avelumab in metastatic PDAC (NCT03193190), with pembrolizumab in gastric cancer (NCT03281369) | |
| Pentoxifylline/treatment for occlusive artery disease | No/yes | None | |
| Pirfenidone/treatment for idiopathic pulmonary fibrosis, TGF-β | No/yes | Phase I with atezolizumab as a second line therapy in NSCL (NCT04467723) |  |
| Tranilast/anti-histamine, TGF-β | No/no (approved as an anti-histamine in Japan and Korea) | None |  |
| Hydralazine/vasodilator | No/yes | None |  |
| Fasudil/vasodilator, Rho-kinase | No/yes | None |  |
| Relaxin/hormone, collagen** | No/No | None |  |
| Halofuginone/antiprotozoal, collagen | No/no (veterinary) | None |  |

* PEGPH20 does not reprogram CAFs, but rather depletes desmoplasia, which has been shown in preclinical studies to induce tumor progression.

**Some studies have demonstrated a correlation between relaxin levels and tumor progression.

For trials in combination with ICB, on April 22, 2022 we searched clinicaltrials.gov for the [name of stromal normalizing therapy] AND pembrolizumab OR atezolizumab OR nivolumab OR cemiplimab OR ipilimumab OR durvalumab OR avelumab OR ipilimumab AND Cancer [DISEASE] with filters for and active or recruiting.

**Supplementary Table 3. Vascular normalizing characteristics of antiangiogenic therapies and immune checkpoint inhibitors.** The efficacy, pharmacodynamic, and adverse effects of the therapies are summarized.

|  | **Antiangiogenic therapy** | **Immune checkpoint inhibition therapy** | **Combination therapy** |
| --- | --- | --- | --- |
| **Anti-tumor efficacy** | Low except in highly angiogenic tumor types such as HCC. | High in some patients | High in patients with tumors that are angiogenic and expressing immune checkpoints.  High in patients with tumors that exclude immune cells and cause immunosuppression through angiogenic signaling. |
| **Vascular pruning** | High. Dependent on dose. | Low in most studies. This could depend on the level of IFNγ generated. | Less than additive. Additional fortification of vessels by the combination could reduce the amount of vessels pruned. |
| **Vascular fortification** | High | High | Could be additive or greater. |
| **Toxicity** | Low | Low | Low |

**List of abbreviations in Supplementary Tables**

| CRC | Colorectal cancer |
| --- | --- |
| CSF1R | Colony stimulating factor 1 receptor |
| CXCL12 | C-X-C motif chemokine ligand 12 |
| CXCR4 | C-X-C chemokine receptor type 4 |
| DDR1 | Discoidin domain receptor 1 |
| DTC | Differentiated thyroid cancers |
| FGFR | Fibroblast growth factor receptor |
| FLT3 | Fms-related tyrosine kinase 3 |
| GI | Gastrointestinal cancer |
| GIST | Gastrointestinal stromal tumor |
| HCC | Hepatocellular carcinoma |
| ICI | Immune checkpoint inhibitor |
| JAK | Janus kinase |
| KIT | v-kit Hardy-Zuckerman 4 feline sarcoma viral oncogene homolog |
| MSI | Microsatellite instability |
| NSCLC | Non-small-cell lung cancer |
| PDAC | Pancreatic ductal adenocarcinoma |
| PDGFR | Platelet-derived growth factor receptor |
| PEGPH20 | Pegvorhyaluronidase alfa |
| PNET | Primitive Neuro-Ectodermal Tumors |
| RCC | Renal cell carcinoma |
| RET | Rearranged during transfection proto-oncogene |
| TGCT | Tenosynovial Giant Cell Tumor |
| TGF- β | Transforming growth factor β |
| TKI | Tyrosine kinase inhibitor |
| TRKA | Tyrosine kinase receptor A |
| TRKC | Tropomyosin receptor kinase C |
| VEGFR | Vascular endothelial growth factor receptor |

**Reference**

1. J. D. Martin, H. Cabral, T. Stylianopoulos, R. K. Jain, Improving cancer immunotherapy using nanomedicine: progress, opportunities and challenges. *Nature Reviews Clinical Oncology* **17**, 251-266 (2020).
